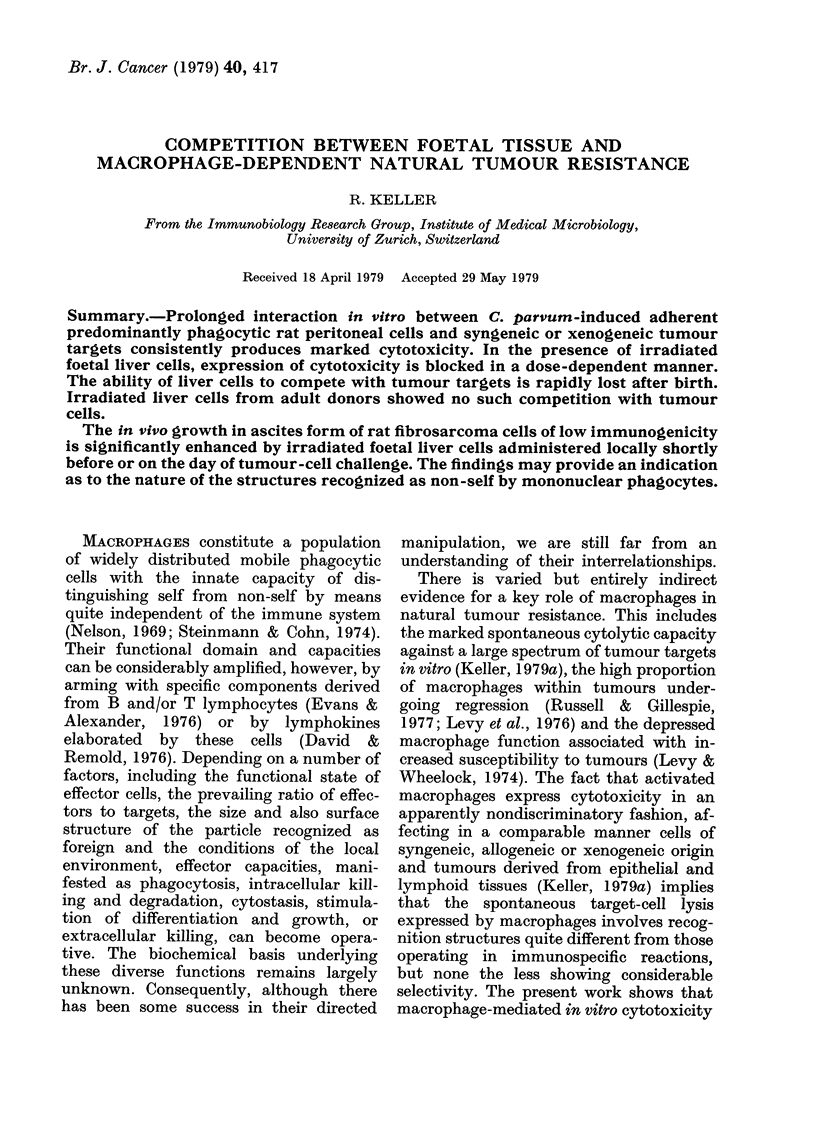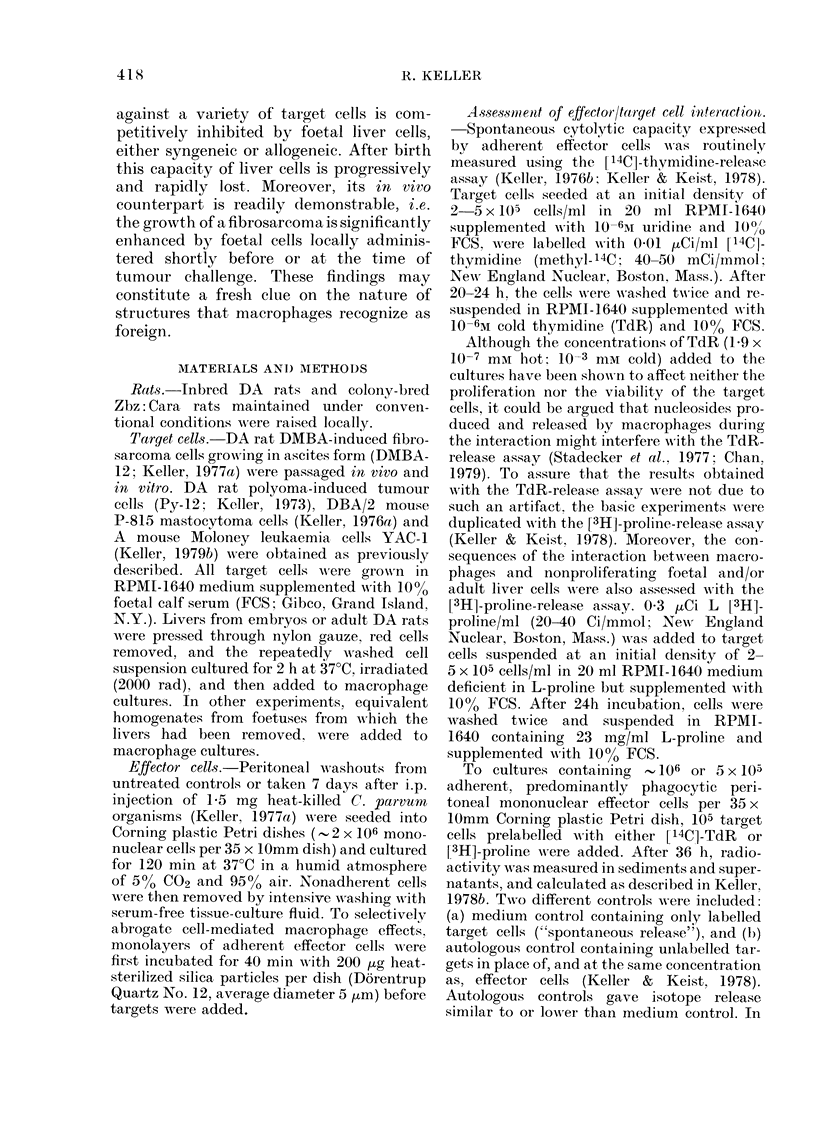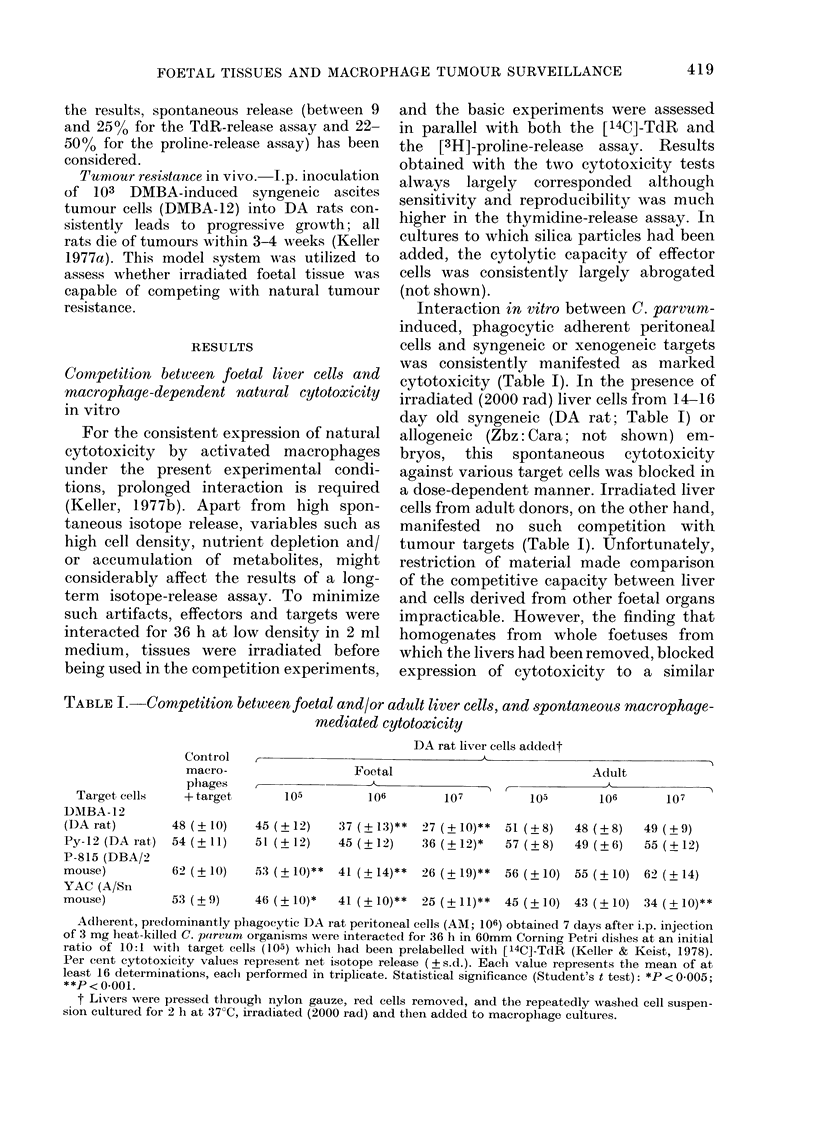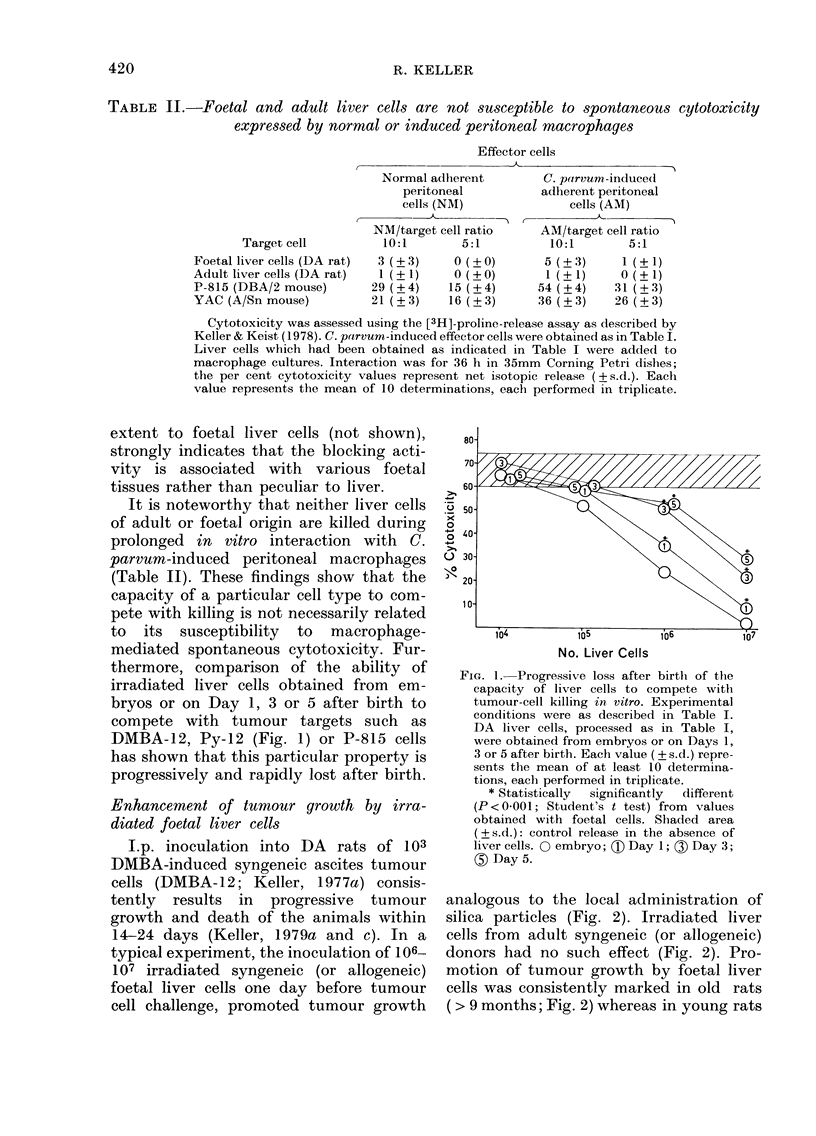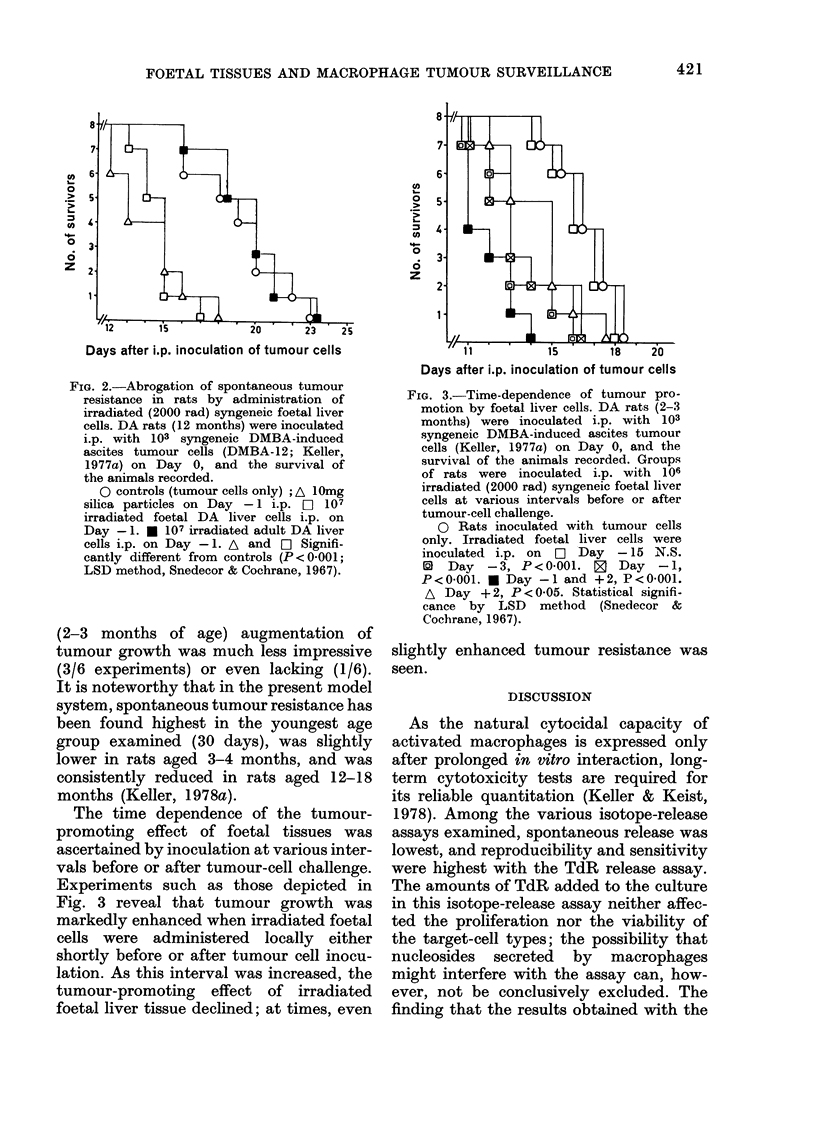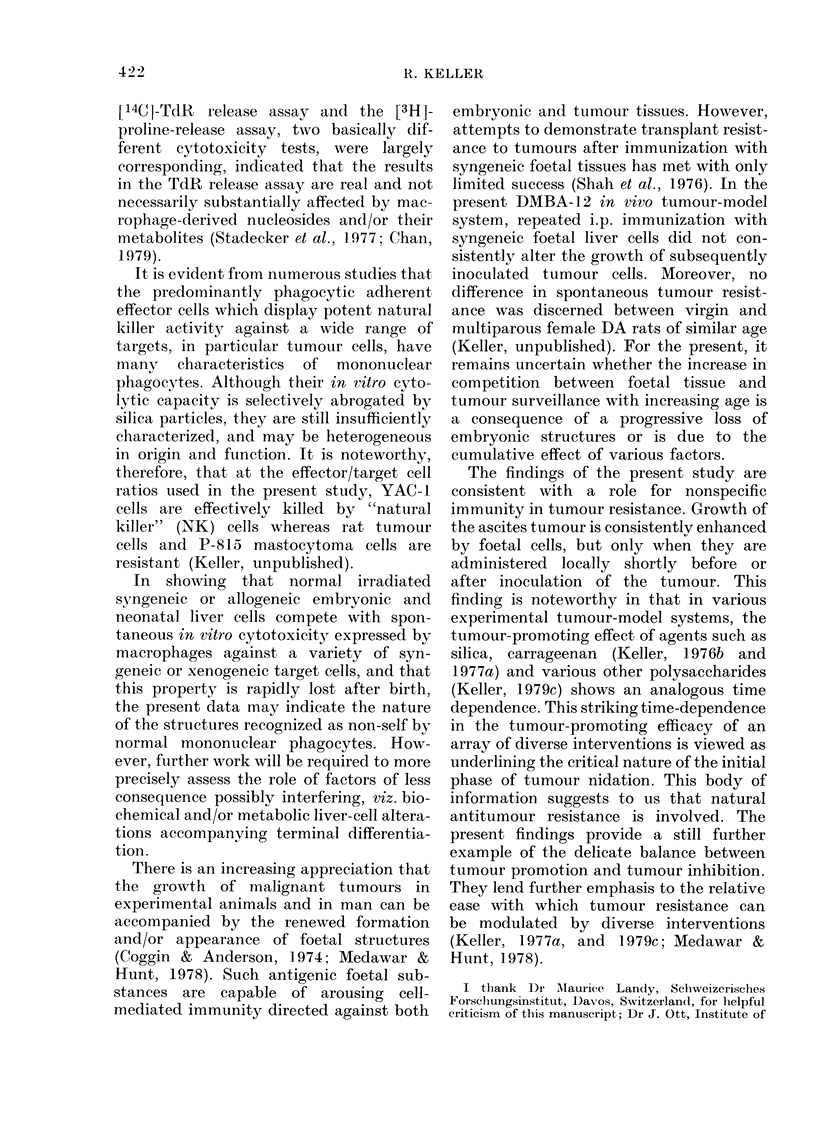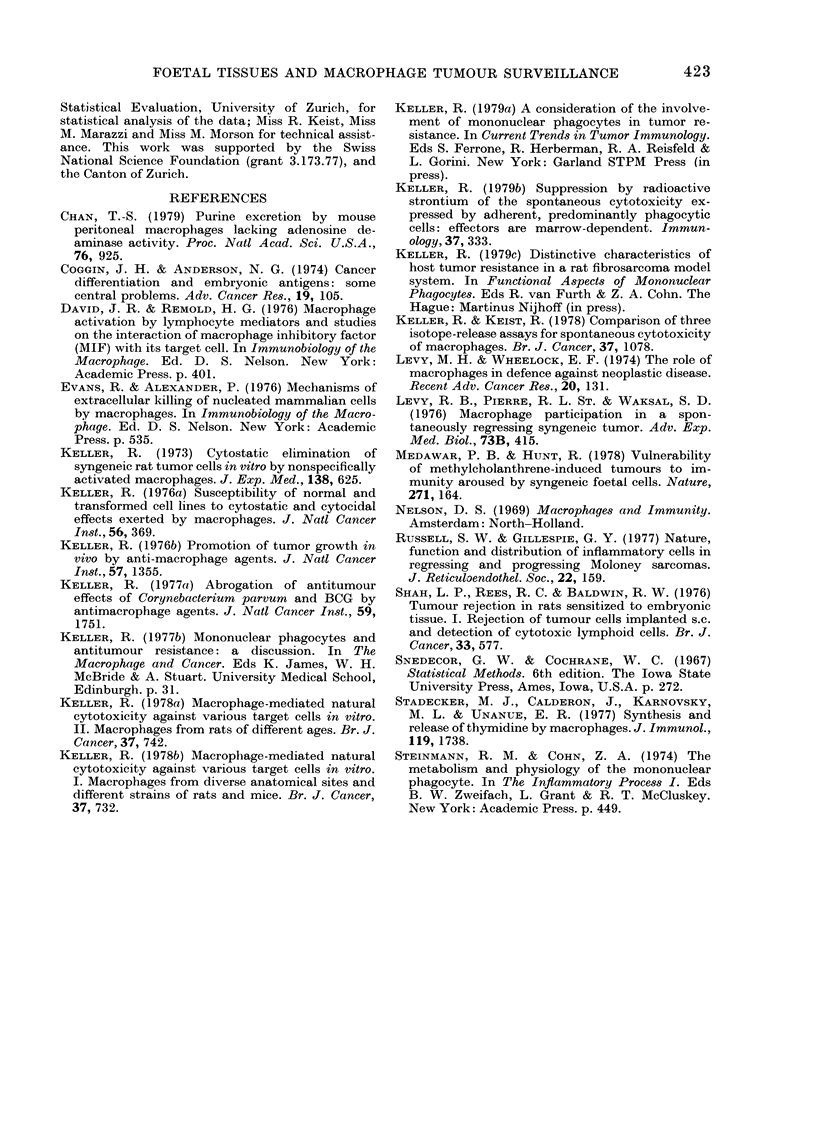# Competition between foetal tissue and macrophage-dependent natural tumour resistance.

**DOI:** 10.1038/bjc.1979.197

**Published:** 1979-09

**Authors:** R. Keller

## Abstract

Prolonged interaction in vitro between C. parvum-induced adherent predominantly phagocytic rat peritoneal cells and syngeneic or xenogeneic tumour targets consistently produces marked cytotoxicity. In the presence of irradiated foetal liver cells, expression of cytotoxicity is blocked in a dose-dependent manner. The ability of liver cells to compete with tumour targets is rapidly lost after birth. Irradiated liver cells from adult donors showed no such competition with tumour cells. The in vivo growth in ascites form of rat fibrosarcoma cells of low immunogenicity is significantly enhanced by irradiated foetal liver cells administered locally shortly before or on the day of tumour-cell challenge. The findings may provide an indication as to the nature of the structures recognized as non-self by mononuclear phagocytes.


*Br. J. Cancer* (1979) **40**, 417

### COMPETITION BETWEEN FOETAL TISSUE AND MACROPHAGE-DEPENDENT NATURAL TUMOUR RESISTANCE

R. KELLER

*From the Immunobiology Research Group, Institute of Medical Microbiology, University of Zurich, Switzerland*

Received 18 April 1979 Accepted 29 May 1979

**Summary.—Prolonged interaction *in vitro* between *C. parvum*-induced adherent predominantly phagocytic rat peritoneal cells and syngeneic or xenogeneic tumour targets consistently produces marked cytotoxicity. In the presence of irradiated foetal liver cells, expression of cytotoxicity is blocked in a dose-dependent manner. The ability of liver cells to compete with tumour targets is rapidly lost after birth. Irradiated liver cells from adult donors showed no such competition with tumour cells.**

**The *in vivo* growth in ascites form of rat fibrosarcoma cells of low immunogenicity is significantly enhanced by irradiated foetal liver cells administered locally shortly before or on the day of tumour-cell challenge. The findings may provide an indication as to the nature of the structures recognized as non-self by mononuclear phagocytes.**

Macrophages constitute a population of widely distributed mobile phagocytic cells with the innate capacity of distinguishing self from non-self by means quite independent of the immune system (Nelson, 1969; Steinmann & Cohn, 1974). Their functional domain and capacities can be considerably amplified, however, by arming with specific components derived from B and/or T lymphocytes (Evans & Alexander, 1976) or by lymphokines elaborated by these cells (David & Remold, 1976). Depending on a number of factors, including the functional state of effector cells, the prevailing ratio of effectors to targets, the size and also surface structure of the particle recognized as foreign and the conditions of the local environment, effector capacities, manifested as phagocytosis, intracellular killing and degradation, cytostasis, stimulation of differentiation and growth, or extracellular killing, can become operative. The biochemical basis underlying these diverse functions remains largely unknown. Consequently, although there has been some success in their directed manipulation, we are still far from an understanding of their interrelationships.

There is varied but entirely indirect evidence for a key role of macrophages in natural tumour resistance. This includes the marked spontaneous cytolytic capacity against a large spectrum of tumour targets *in vitro* (Keller, 1979*a*), the high proportion of macrophages within tumours undergoing regression (Russell & Gillespie, 1977; Levy *et al.*, 1976) and the depressed macrophage function associated with increased susceptibility to tumours (Levy & Wheelock, 1974). The fact that activated macrophages express cytotoxicity in an apparently nondiscriminatory fashion, affecting in a comparable manner cells of syngeneic, allogeneic or xenogeneic origin and tumours derived from epithelial and lymphoid tissues (Keller, 1979*a*) implies that the spontaneous target-cell lysis expressed by macrophages involves recognition structures quite different from those operating in immunospecific reactions, but none the less showing considerable selectivity. The present work shows that macrophage-mediated *in vitro* cytotoxicity against a variety of target cells is competitively inhibited by foetal liver cells, either syngeneic or allogeneic. After birth this capacity of liver cells is progressively and rapidly lost. Moreover, its *in vivo* counterpart is readily demonstrable, *i.e.* the growth of a fibrosarcoma is significantly enhanced by foetal cells locally administered shortly before or at the time of tumour challenge. These findings may constitute a fresh clue on the nature of structures that macrophages recognize as foreign.

### MATERIALS AND METHODS

*Rats.*—Inbred DA rats and colony-bred Zbz:Cara rats maintained under conventional conditions were raised locally.

*Target cells.*—DA rat DMBA-induced fibrosarcoma cells growing in ascites form (DMBA-12; Keller, 1977*a*) were passaged *in vivo* and *in vitro.* DA rat polyoma-induced tumour cells (Py-12; Keller, 1973), DBA/2 mouse P-815 mastocytoma cells (Keller, 1976*a*) and A mouse Moloney leukaemia cells YAC-1 (Keller, 1979*b*) were obtained as previously described. All target cells were grown in RPMI-1640 medium supplemented with 10% foetal calf serum (FCS; Gibco, Grand Island, N.Y.). Livers from embryos or adult DA rats were pressed through nylon gauze, red cells removed, and the repeatedly washed cell suspension cultured for 2 h at 37°C, irradiated (2000 rad), and then added to macrophage cultures. In other experiments, equivalent homogenates from foetuses from which the livers had been removed, were added to macrophage cultures.

*Effector cells.*—Peritoneal washouts from untreated controls or taken 7 days after i.p. injection of 1·5 mg heat-killed *C. parvum* organisms (Keller, 1977*a*) were seeded into Corning plastic Petri dishes ($\sim 2 \times 10^6$ mononuclear cells per 35 x 10mm dish) and cultured for 120 min at 37°C in a humid atmosphere of 5% $CO_2$ and 95% air. Nonadherent cells were then removed by intensive washing with serum-free tissue-culture fluid. To selectively abrogate cell-mediated macrophage effects, monolayers of adherent effector cells were first incubated for 40 min with 200 μg heat-sterilized silica particles per dish (Dörentrup Quartz No. 12, average diameter 5 μm) before targets were added.

*Assessment of effector/target cell interaction.*—Spontaneous cytolytic capacity expressed by adherent effector cells was routinely measured using the [$^{14}$C]-thymidine-release assay (Keller, 1976*b*; Keller & Keist, 1978). Target cells seeded at an initial density of 2—$5 \times 10^5$ cells/ml in 20 ml RPMI-1640 supplemented with $10^{-6}$M uridine and 10% FCS, were labelled with 0·01 μCi/ml [$^{14}$C]-thymidine (methyl-$^{14}$C; 40–50 mCi/mmol; New England Nuclear, Boston, Mass.). After 20–24 h, the cells were washed twice and resuspended in RPMI-1640 supplemented with $10^{-6}$M cold thymidine (TdR) and 10% FCS.

Although the concentrations of TdR ($1{\cdot}9 \times 10^{-7}$ mM hot; $10^{-3}$ mM cold) added to the cultures have been shown to affect neither the proliferation nor the viability of the target cells, it could be argued that nucleosides produced and released by macrophages during the interaction might interfere with the TdR-release assay (Stadecker *et al.*, 1977; Chan, 1979). To assure that the results obtained with the TdR-release assay were not due to such an artifact, the basic experiments were duplicated with the [$^3$H]-proline-release assay (Keller & Keist, 1978). Moreover, the consequences of the interaction between macrophages and nonproliferating foetal and/or adult liver cells were also assessed with the [$^3$H]-proline-release assay. 0·3 μCi L [$^3$H]-proline/ml (20–40 Ci/mmol; New England Nuclear, Boston, Mass.) was added to target cells suspended at an initial density of 2–$5 \times 10^5$ cells/ml in 20 ml RPMI-1640 medium deficient in L-proline but supplemented with 10% FCS. After 24h incubation, cells were washed twice and suspended in RPMI-1640 containing 23 mg/ml L-proline and supplemented with 10% FCS.

To cultures containing $\sim 10^6$ or $5 \times 10^5$ adherent, predominantly phagocytic peritoneal mononuclear effector cells per 35 x 10mm Corning plastic Petri dish, $10^5$ target cells prelabelled with either [$^{14}$C]-TdR or [$^3$H]-proline were added. After 36 h, radioactivity was measured in sediments and supernatants, and calculated as described in Keller, 1978*b*. Two different controls were included: (a) medium control containing only labelled target cells ("spontaneous release"), and (b) autologous control containing unlabelled targets in place of, and at the same concentration as, effector cells (Keller & Keist, 1978). Autologous controls gave isotope release similar to or lower than medium control. In the results, spontaneous release (between 9 and 25% for the TdR-release assay and 22–50% for the proline-release assay) has been considered.

*Tumour resistance* in vivo.—I.p. inoculation of $10^3$ DMBA-induced syngeneic ascites tumour cells (DMBA-12) into DA rats consistently leads to progressive growth; all rats die of tumours within 3–4 weeks (Keller 1977*a*). This model system was utilized to assess whether irradiated foetal tissue was capable of competing with natural tumour resistance.

### RESULTS

### *Competition between foetal liver cells and macrophage-dependent natural cytotoxicity* in vitro

For the consistent expression of natural cytotoxicity by activated macrophages under the present experimental conditions, prolonged interaction is required (Keller, 1977b). Apart from high spontaneous isotope release, variables such as high cell density, nutrient depletion and/or accumulation of metabolites, might considerably affect the results of a long-term isotope-release assay. To minimize such artifacts, effectors and targets were interacted for 36 h at low density in 2 ml medium, tissues were irradiated before being used in the competition experiments, and the basic experiments were assessed in parallel with both the [$^{14}$C]-TdR and the [$^3$H]-proline-release assay. Results obtained with the two cytotoxicity tests always largely corresponded although sensitivity and reproducibility was much higher in the thymidine-release assay. In cultures to which silica particles had been added, the cytolytic capacity of effector cells was consistently largely abrogated (not shown).

Interaction *in vitro* between *C. parvum*-induced, phagocytic adherent peritoneal cells and syngeneic or xenogeneic targets was consistently manifested as marked cytotoxicity (Table I). In the presence of irradiated (2000 rad) liver cells from 14–16 day old syngeneic (DA rat; Table I) or allogeneic (Zbz:Cara; not shown) embryos, this spontaneous cytotoxicity against various target cells was blocked in a dose-dependent manner. Irradiated liver cells from adult donors, on the other hand, manifested no such competition with tumour targets (Table I). Unfortunately, restriction of material made comparison of the competitive capacity between liver and cells derived from other foetal organs impracticable. However, the finding that homogenates from whole foetuses from which the livers had been removed, blocked expression of cytotoxicity to a similar

TABLE I.—*Competition between foetal and/or adult liver cells, and spontaneous macrophage-mediated cytotoxicity*

| Target cells | Control macro-phages + target | DA rat liver cells added† Foetal $10^5$ | Foetal $10^6$ | Foetal $10^7$ | Adult $10^5$ | Adult $10^6$ | Adult $10^7$ |
|---|---|---|---|---|---|---|---|
| DMBA-12 (DA rat) | 48 (±10) | 45 (±12) | 37 (±13)** | 27 (±10)** | 51 (±8) | 48 (±8) | 49 (±9) |
| Py-12 (DA rat) | 54 (±11) | 51 (±12) | 45 (±12) | 36 (±12)* | 57 (±8) | 49 (±6) | 55 (±12) |
| P-815 (DBA/2 mouse) | 62 (±10) | 53 (±10)** | 41 (±14)** | 26 (±19)** | 56 (±10) | 55 (±10) | 62 (±14) |
| YAC (A/Sn mouse) | 53 (±9) | 46 (±10)* | 41 (±10)** | 25 (±11)** | 45 (±10) | 43 (±10) | 34 (±10)** |

Adherent, predominantly phagocytic DA rat peritoneal cells (AM; $10^6$) obtained 7 days after i.p. injection of 3 mg heat-killed *C. parvum* organisms were interacted for 36 h in 60mm Corning Petri dishes at an initial ratio of 10:1 with target cells ($10^5$) which had been prelabelled with [$^{14}$C]-TdR (Keller & Keist, 1978). Per cent cytotoxicity values represent net isotope release (±s.d.). Each value represents the mean of at least 16 determinations, each performed in triplicate. Statistical significance (Student's *t* test): $*P < 0{\cdot}005$; $**P < 0{\cdot}001$.

† Livers were pressed through nylon gauze, red cells removed, and the repeatedly washed cell suspension cultured for 2 h at 37°C, irradiated (2000 rad) and then added to macrophage cultures.

TABLE II.—*Foetal and adult liver cells are not susceptible to spontaneous cytotoxicity expressed by normal or induced peritoneal macrophages*

| | Effector cells | | | |
|---|---|---|---|---|
| | Normal adherent peritoneal cells (NM) | | *C. parvum*-induced adherent peritoneal cells (AM) | |
| | NM/target cell ratio | | AM/target cell ratio | |
| Target cell | 10:1 | 5:1 | 10:1 | 5:1 |
| Foetal liver cells (DA rat) | 3 (±3) | 0 (±0) | 5 (±3) | 1 (±1) |
| Adult liver cells (DA rat) | 1 (±1) | 0 (±0) | 1 (±1) | 0 (±1) |
| P-815 (DBA/2 mouse) | 29 (±4) | 15 (±4) | 54 (±4) | 31 (±3) |
| YAC (A/Sn mouse) | 21 (±3) | 16 (±3) | 36 (±3) | 26 (±3) |

Cytotoxicity was assessed using the [$^3$H]-proline-release assay as described by Keller & Keist (1978). *C. parvum*-induced effector cells were obtained as in Table I. Liver cells which had been obtained as indicated in Table I were added to macrophage cultures. Interaction was for 36 h in 35mm Corning Petri dishes; the per cent cytotoxicity values represent net isotopic release (±s.d.). Each value represents the mean of 10 determinations, each performed in triplicate.

extent to foetal liver cells (not shown), strongly indicates that the blocking activity is associated with various foetal tissues rather than peculiar to liver.

It is noteworthy that neither liver cells of adult or foetal origin are killed during prolonged *in vitro* interaction with *C. parvum*-induced peritoneal macrophages (Table II). These findings show that the capacity of a particular cell type to compete with killing is not necessarily related to its susceptibility to macrophage-mediated spontaneous cytotoxicity. Furthermore, comparison of the ability of irradiated liver cells obtained from embryos or on Day 1, 3 or 5 after birth to compete with tumour targets such as DMBA-12, Py-12 (Fig. 1) or P-815 cells has shown that this particular property is progressively and rapidly lost after birth.

FIG. 1.—Progressive loss after birth of the capacity of liver cells to compete with tumour-cell killing *in vitro*. Experimental conditions were as described in Table I. DA liver cells, processed as in Table I, were obtained from embryos or on Days 1, 3 or 5 after birth. Each value (±s.d.) represents the mean of at least 10 determinations, each performed in triplicate.

* Statistically significantly different ($P < 0.001$; Student's $t$ test) from values obtained with foetal cells. Shaded area (±s.d.): control release in the absence of liver cells. ○ embryo; ① Day 1; ③ Day 3; ⑤ Day 5.

*Enhancement of tumour growth by irradiated foetal liver cells*

I.p. inoculation into DA rats of $10^3$ DMBA-induced syngeneic ascites tumour cells (DMBA-12; Keller, 1977*a*) consistently results in progressive tumour growth and death of the animals within 14–24 days (Keller, 1979*a* and *c*). In a typical experiment, the inoculation of $10^6$–$10^7$ irradiated syngeneic (or allogeneic) foetal liver cells one day before tumour cell challenge, promoted tumour growth analogous to the local administration of silica particles (Fig. 2). Irradiated liver cells from adult syngeneic (or allogeneic) donors had no such effect (Fig. 2). Promotion of tumour growth by foetal liver cells was consistently marked in old rats (> 9 months; Fig. 2) whereas in young rats

FIG. 2.—Abrogation of spontaneous tumour resistance in rats by administration of irradiated (2000 rad) syngeneic foetal liver cells. DA rats (12 months) were inoculated i.p. with $10^3$ syngeneic DMBA-induced ascites tumour cells (DMBA-12; Keller, 1977*a*) on Day 0, and the survival of the animals recorded.

○ controls (tumour cells only) ; △ 10mg silica particles on Day −1 i.p. □ $10^7$ irradiated foetal DA liver cells i.p. on Day −1. ■ $10^7$ irradiated adult DA liver cells i.p. on Day −1. △ and □ Significantly different from controls ($P < 0{\cdot}001$; LSD method, Snedecor & Cochrane, 1967).

FIG. 3.—Time-dependence of tumour promotion by foetal liver cells. DA rats (2–3 months) were inoculated i.p. with $10^3$ syngeneic DMBA-induced ascites tumour cells (Keller, 1977*a*) on Day 0, and the survival of the animals recorded. Groups of rats were inoculated i.p. with $10^6$ irradiated (2000 rad) syngeneic foetal liver cells at various intervals before or after tumour-cell challenge.

○ Rats inoculated with tumour cells only. Irradiated foetal liver cells were inoculated i.p. on □ Day −15 N.S. ▣ Day −3, $P < 0{\cdot}001$. ⊠ Day −1, $P < 0{\cdot}001$. ■ Day −1 and +2, $P < 0{\cdot}001$. △ Day +2, $P < 0{\cdot}05$. Statistical significance by LSD method (Snedecor & Cochrane, 1967).

(2–3 months of age) augmentation of tumour growth was much less impressive (3/6 experiments) or even lacking (1/6). It is noteworthy that in the present model system, spontaneous tumour resistance has been found highest in the youngest age group examined (30 days), was slightly lower in rats aged 3–4 months, and was consistently reduced in rats aged 12–18 months (Keller, 1978*a*).

The time dependence of the tumour-promoting effect of foetal tissues was ascertained by inoculation at various intervals before or after tumour-cell challenge. Experiments such as those depicted in Fig. 3 reveal that tumour growth was markedly enhanced when irradiated foetal cells were administered locally either shortly before or after tumour cell inoculation. As this interval was increased, the tumour-promoting effect of irradiated foetal liver tissue declined; at times, even slightly enhanced tumour resistance was seen.

### DISCUSSION

As the natural cytocidal capacity of activated macrophages is expressed only after prolonged *in vitro* interaction, long-term cytotoxicity tests are required for its reliable quantitation (Keller & Keist, 1978). Among the various isotope-release assays examined, spontaneous release was lowest, and reproducibility and sensitivity were highest with the TdR release assay. The amounts of TdR added to the culture in this isotope-release assay neither affected the proliferation nor the viability of the target-cell types; the possibility that nucleosides secreted by macrophages might interfere with the assay can, however, not be conclusively excluded. The finding that the results obtained with the

[$^{14}$C]-TdR release assay and the [$^{3}$H]-proline-release assay, two basically different cytotoxicity tests, were largely corresponding, indicated that the results in the TdR release assay are real and not necessarily substantially affected by macrophage-derived nucleosides and/or their metabolites (Stadecker *et al.*, 1977; Chan, 1979).

It is evident from numerous studies that the predominantly phagocytic adherent effector cells which display potent natural killer activity against a wide range of targets, in particular tumour cells, have many characteristics of mononuclear phagocytes. Although their *in vitro* cytolytic capacity is selectively abrogated by silica particles, they are still insufficiently characterized, and may be heterogeneous in origin and function. It is noteworthy, therefore, that at the effector/target cell ratios used in the present study, YAC-1 cells are effectively killed by "natural killer" (NK) cells whereas rat tumour cells and P-815 mastocytoma cells are resistant (Keller, unpublished).

In showing that normal irradiated syngeneic or allogeneic embryonic and neonatal liver cells compete with spontaneous *in vitro* cytotoxicity expressed by macrophages against a variety of syngeneic or xenogeneic target cells, and that this property is rapidly lost after birth, the present data may indicate the nature of the structures recognized as non-self by normal mononuclear phagocytes. However, further work will be required to more precisely assess the role of factors of less consequence possibly interfering, *viz.* biochemical and/or metabolic liver-cell alterations accompanying terminal differentiation.

There is an increasing appreciation that the growth of malignant tumours in experimental animals and in man can be accompanied by the renewed formation and/or appearance of foetal structures (Coggin & Anderson, 1974; Medawar & Hunt, 1978). Such antigenic foetal substances are capable of arousing cell-mediated immunity directed against both embryonic and tumour tissues. However, attempts to demonstrate transplant resistance to tumours after immunization with syngeneic foetal tissues has met with only limited success (Shah *et al.*, 1976). In the present DMBA-12 *in vivo* tumour-model system, repeated i.p. immunization with syngeneic foetal liver cells did not consistently alter the growth of subsequently inoculated tumour cells. Moreover, no difference in spontaneous tumour resistance was discerned between virgin and multiparous female DA rats of similar age (Keller, unpublished). For the present, it remains uncertain whether the increase in competition between foetal tissue and tumour surveillance with increasing age is a consequence of a progressive loss of embryonic structures or is due to the cumulative effect of various factors.

The findings of the present study are consistent with a role for nonspecific immunity in tumour resistance. Growth of the ascites tumour is consistently enhanced by foetal cells, but only when they are administered locally shortly before or after inoculation of the tumour. This finding is noteworthy in that in various experimental tumour-model systems, the tumour-promoting effect of agents such as silica, carrageenan (Keller, 1976*b* and 1977*a*) and various other polysaccharides (Keller, 1979*c*) shows an analogous time dependence. This striking time-dependence in the tumour-promoting efficacy of an array of diverse interventions is viewed as underlining the critical nature of the initial phase of tumour nidation. This body of information suggests to us that natural antitumour resistance is involved. The present findings provide a still further example of the delicate balance between tumour promotion and tumour inhibition. They lend further emphasis to the relative ease with which tumour resistance can be modulated by diverse interventions (Keller, 1977*a*, and 1979*c*; Medawar & Hunt, 1978).

I thank Dr Maurice Landy, Schweizerisches Forschungsinstitut, Davos, Switzerland, for helpful criticism of this manuscript; Dr J. Ott, Institute of

Statistical Evaluation, University of Zurich, for statistical analysis of the data; Miss R. Keist, Miss M. Marazzi and Miss M. Morson for technical assistance. This work was supported by the Swiss National Science Foundation (grant 3.173.77), and the Canton of Zurich.

### REFERENCES

CHAN, T.-S. (1979) Purine excretion by mouse peritoneal macrophages lacking adenosine deaminase activity. *Proc. Natl Acad. Sci. U.S.A.*, **76**, 925.

COGGIN, J. H. & ANDERSON, N. G. (1974) Cancer differentiation and embryonic antigens: some central problems. *Adv. Cancer Res.*, **19**, 105.

DAVID, J. R. & REMOLD, H. G. (1976) Macrophage activation by lymphocyte mediators and studies on the interaction of macrophage inhibitory factor (MIF) with its target cell. In *Immunobiology of the Macrophage*. Ed. D. S. Nelson. New York: Academic Press. p. 401.

EVANS, R. & ALEXANDER, P. (1976) Mechanisms of extracellular killing of nucleated mammalian cells by macrophages. In *Immunobiology of the Macrophage*. Ed. D. S. Nelson. New York: Academic Press. p. 535.

KELLER, R. (1973) Cytostatic elimination of syngeneic rat tumor cells *in vitro* by nonspecifically activated macrophages. *J. Exp. Med.*, **138**, 625.

KELLER, R. (1976*a*) Susceptibility of normal and transformed cell lines to cytostatic and cytocidal effects exerted by macrophages. *J. Natl Cancer Inst.*, **56**, 369.

KELLER, R. (1976*b*) Promotion of tumor growth *in vivo* by anti-macrophage agents. *J. Natl Cancer Inst.*, **57**, 1355.

KELLER, R. (1977*a*) Abrogation of antitumour effects of *Corynebacterium parvum* and BCG by antimacrophage agents. *J. Natl Cancer Inst.*, **59**, 1751.

KELLER, R. (1977*b*) Mononuclear phagocytes and antitumour resistance: a discussion. In *The Macrophage and Cancer*. Eds K. James, W. H. McBride & A. Stuart. University Medical School, Edinburgh. p. 31.

KELLER, R. (1978*a*) Macrophage-mediated natural cytotoxicity against various target cells *in vitro*. II. Macrophages from rats of different ages. *Br. J. Cancer*, **37**, 742.

KELLER, R. (1978*b*) Macrophage-mediated natural cytotoxicity against various target cells *in vitro*. I. Macrophages from diverse anatomical sites and different strains of rats and mice. *Br. J. Cancer*, **37**, 732.

KELLER, R. (1979*a*) A consideration of the involvement of mononuclear phagocytes in tumor resistance. In *Current Trends in Tumor Immunology*. Eds S. Ferrone, R. Herberman, R. A. Reisfeld & L. Gorini. New York: Garland STPM Press (in press).

KELLER, R. (1979*b*) Suppression by radioactive strontium of the spontaneous cytotoxicity expressed by adherent, predominantly phagocytic cells: effectors are marrow-dependent. *Immunology*, **37**, 333.

KELLER, R. (1979*c*) Distinctive characteristics of host tumor resistance in a rat fibrosarcoma model system. In *Functional Aspects of Mononuclear Phagocytes*. Eds R. van Furth & Z. A. Cohn. The Hague: Martinus Nijhoff (in press).

KELLER, R. & KEIST, R. (1978) Comparison of three isotope-release assays for spontaneous cytotoxicity of macrophages. *Br. J. Cancer*, **37**, 1078.

LEVY, M. H. & WHEELOCK, E. F. (1974) The role of macrophages in defence against neoplastic disease. *Recent Adv. Cancer Res.*, **20**, 131.

LEVY, R. B., PIERRE, R. L. ST. & WAKSAL, S. D. (1976) Macrophage participation in a spontaneously regressing syngeneic tumor. *Adv. Exp. Med. Biol.*, **73B**, 415.

MEDAWAR, P. B. & HUNT, R. (1978) Vulnerability of methylcholanthrene-induced tumours to immunity aroused by syngeneic foetal cells. *Nature*, **271**, 164.

NELSON, D. S. (1969) *Macrophages and Immunity*. Amsterdam: North-Holland.

RUSSELL, S. W. & GILLESPIE, G. Y. (1977) Nature, function and distribution of inflammatory cells in regressing and progressing Moloney sarcomas. *J. Reticuloendothel. Soc.*, **22**, 159.

SHAH, L. P., REES, R. C. & BALDWIN, R. W. (1976) Tumour rejection in rats sensitized to embryonic tissue. I. Rejection of tumour cells implanted s.c. and detection of cytotoxic lymphoid cells. *Br. J. Cancer*, **33**, 577.

SNEDECOR, G. W. & COCHRANE, W. C. (1967) *Statistical Methods*. 6th edition. The Iowa State University Press, Ames, Iowa, U.S.A. p. 272.

STADECKER, M. J., CALDERON, J., KARNOVSKY, M. L. & UNANUE, E. R. (1977) Synthesis and release of thymidine by macrophages. *J. Immunol.*, **119**, 1738.

STEINMANN, R. M. & COHN, Z. A. (1974) The metabolism and physiology of the mononuclear phagocyte. In *The Inflammatory Process I*. Eds B. W. Zweifach, L. Grant & R. T. McCluskey. New York: Academic Press. p. 449.